# Action Observation Combined With Conventional Training Improves the Rugby Lineout Throwing Performance: A Pilot Study

**DOI:** 10.3389/fpsyg.2019.00889

**Published:** 2019-04-24

**Authors:** Emanuela Faelli, Laura Strassera, Elisa Pelosin, Luisa Perasso, Vittoria Ferrando, Ambra Bisio, Piero Ruggeri

**Affiliations:** ^1^Department of Experimental Medicine, Section of Human Physiology, University of Genoa, Genoa, Italy; ^2^Centro Polifunzionale di Scienze Motorie, University of Genoa, Genoa, Italy; ^3^Department of Neuroscience, Rehabilitation, Ophthalmology, Genetics, Maternal, and Child Health, University of Genoa, Genoa, Italy; ^4^Ospedale Policlinico San Martino, IRCCS, Genoa, Italy

**Keywords:** action observation, lineout throw, sport training, rugby, kinematics

## Abstract

Combining action observation (AO) and physical practice contributes to motor skill learning, and a number of studies pointed out the beneficial role of AO training in improving the motor performance and the athletes' movement kinematics. The aim of this study was to investigate if AO combined with immediate conventional training was able to improve motor performance and kinematic parameters of a complex motor skill such as the lineout throw, a gesture that represents a key aspect of rugby, that is unique to this sport. Twenty elite rugby players were divided into two groups. The AO group watched a 5-min video-clip of an expert model performing the lineout throw toward a target at 7 m distance and, immediately after the AO, this group executed the conventional training, consisting of six repetitions x five blocks of throws. The CONTROL group performed only the conventional lineout training. Intervention period lasted 4 weeks, 3 sessions/week. The AO group showed significant improvements in throwing accuracy (i.e., number of throws hitting the target), whilst no significant changes were observed in the CONTROL group. As concerns kinematic parameters, hooker's arm mean velocity significantly increased in both groups, but the increase was higher in AO group compared to CONTROL group. Ball velocity significantly increased only in the AO group, whereas ball angle release and ball spinning significantly decreased in both groups, with no differences between groups. Finally, no significant changes in knee and elbow angles were observed. Our results showed that the combination of AO and conventional training was more effective than a conventional training alone in improving the performance of elite rugby players, in executing a complex motor skill, such as the lineout. This combined training led to significant improvements in throwing accuracy and in hooker's and ball's kinematic parameters. Since AO can be easily implemented in combination with conventional training, the results of this study can encourage coaches in designing specific lineout training programs, which include AO cognitive training.

## Introduction

The learning of a motor skill is commonly achieved through physical practice (e.g., task repetition), but recent studies have demonstrated that training employing action observation (AO) and/or motor imagery in combination with movement execution, is able to facilitate skill learning and to improve motor performance (Hodges and Williams, 2012). Behind these results there is a huge scientific literature showing the activation of a brain network, the mirror neurons system (MNS) that activates during action execution, AO and motor imagery (Fadiga et al., 1995, 1999). Indeed, a number of studies proposed that AO and motor imagery are functionally equivalent to action execution and rely on common neural structures which include motor-related brain areas (e.g., Lotze et al., 1999; Grèzes and Decety, 2001; Jeannerod, 2001; Clark et al., 2004; Filimon et al., 2007; Holmes and Calmels, 2008; Lorey et al., 2013; Zabicki et al., 2017). It was shown that these activations depend on the visual perspective of the observers (e.g., Maeda et al., 2002), on the imagery modality (e.g., Seiler et al., 2015) and is related to the person's motor experience (Bisio et al., 2010; Avanzino et al., 2015; Lagravinese et al., 2017). Particularly interesting is the example provided by Calvo-Merino et al. (2005) on the motor expertise in different dance styles. Two groups of subjects, expert ballet, and capoeira dancers, watched videos of these dance styles (ballet and capoeira) during a functional magnetic resonance imaging examination. Results showed that the brain's response during video observation increased when the observed movement belonged to the participant's motor repertoire. Furthermore, in order to rule out the possibility that this effect was due to a mere visual familiarity with the observed action and not to the motor repertoire, the same research group showed to male and female dancers videos of gender-specific male and female ballet moves (Calvo-Merino et al., 2006). Results revealed that brain's region associated to the mirror neuron system have a purely motor response over and above visual representations of action. Nevertheless, when explicitly assessing the role of visual experience with a certain dance style with respect to others, the results of Jola et al. (2012) showed that even without physical training, corticospinal excitability is modulated as a function of the visual familiarity. Similar results were obtained when the AO network activity was investigated when an athlete observed a movement belonging to a sport in which she/he was expert or not (Wright et al., 2010, 2011; Abreu et al., 2012; Bishop et al., 2013; Balser et al., 2014a,b). All these studies pointed out the strict link between AO and action execution, and for this reason AO was proposed as possible fruitful methodology to enhance the results obtained via conventional motor learning techniques.

In sport settings, expert model demonstrations are widely used by instructors as a teaching technique to facilitate the acquisition of new motor patterns. However, the efficacy of this technique in the different sport fields has still to be demonstrated. Observational training was shown to enhance motor performance (Ste-Marie et al., 2012), movement coordination (Horn et al., 2007), and to promote the acquisition of new motor skills (Hodges et al., 2007). Other studies revealed that kinematic parameters changed toward those of the model, leading to a better motor performance (Hayes et al., 2007; Hodges et al., 2007). A number of studies demonstrated a facilitation of learning when physical practice is combined with observational learning. One of the first study on this matter was published by Magill and Schoenfelder-Zohdi (1996), who asked rhythmic gymnastics athletes to observe the video of an expert model and reported the positive effects on performance. Later, it was shown that kinematic patterns in soccer players (Hodges et al., 2005), cricket (Breslin et al., 2006), bowling (Hayes et al., 2007), and weightlifting (Sakadjian et al., 2014) improved after the combination of AO and physical practice more than physical practice alone. During motor learning processes, AO facilitated learning of motor skills, and increased the coordination pattern of the limbs in term of intra-limb relative motion (angular value and its variability) at level of the upper and lower limbs (Breslin et al., 2006; Horn et al., 2007). Furthermore, the combination of AO and physical practice has been shown to potentiate the motor performance in sports that implicate the use of a ball, like volleyball (Weeks and Anderson, 2000) and golf (Guadagnoli et al., 2002; Kim et al., 2011; D'Innocenzo et al., 2016; Nishizawa and Kimura, 2017). For instance, Weeks and Anderson (2000) investigated the acquisition and the retention of a sport-specific motor skill in volleyball players, showing the benefits of AO in learning and retaining this skill for novice individuals. Indeed, the expert model provided a correct example of the specific movement pattern, giving to the observer the information regarding the best strategy to perform the motor performance (Rohbanfard and Proteau, 2011).

Furthermore, the efficacy of AO was tested also on another aspects of the model's movement kinematics, namely the temporal feature of the observed action (Gavazzi et al., 2013). Particularly, it was demonstrated that the visual demonstration facilitated the identification of specific movement strategies leading the observer to mimic the expert model's velocity (Al-Abood et al., 2001). Further, it was shown that video instructions improved motor performance in term of movement accuracy, when compared to other techniques not involving AO (Guadagnoli et al., 2002).

All together, these results posit the benefits of the AO in sports, as this technique might affect both movement kinematics and performance accuracy. Therefore, the combination of AO and physical practice might be more effective than physical practice alone in improving the motor performance, in terms of changes in performance accuracy and movement kinematics, on specific motor skills.

An interesting example of a complex motor skill, whose performance accuracy depends on both movement kinematic and temporal features is the lineout throw in rugby union. The rugby lineout is a key aspect of game play that is unique to the sport. It is used to restart the play after the ball has crossed the side-line of the field. The lineout throw is executed by one player, the hooker, who throws the ball toward units of jumpers and lifters, who attempt to intercept the ball in order to resume the game.

One of the fundamental factor for a successful lineout is an accurate throw (Trewartha et al., 2008). The lineout throwing action adopted by most players is a two-handed overhead delivery with the ball being spun about its longitudinal axis during flight. Lineout throws travels approximately between 5 and 7 m or 15 and 18 m in length and 3–3.5 m above the ground, in a straight line, perpendicular to the touchline (World Rugby (ed), 2016). It is usually divided into two phases: backswing and forward swing. The backswing phase starts at initiation of the backward ball movement from the thrower's set position and ends when the ball stopped moving backwards, whilst the forward swing starts at the completion of the backswing and continues through until ball release (Sayers, 2011).

Only few studies on the biomechanical analyses of lineout throwing are present in scientific literature (Sayers, 2005, 2011; Trewartha et al., 2008; Croft et al., 2012). Overall, these researches highlight the importance to work on movement kinematics, in term of hooker's joints angles and movement lineout throwing velocity, in order to improve the outcome of the motor performance, that can be evaluated by the throwing accuracy.

The aim of this study was to investigate in elite rugby players whether a combination of AO and conventional training is able to improve throwing technique of the lineout and the outcome of the motor performance, more than conventional training alone. The number of throws hitting a target was used to evaluate the outcome of the motor performance, whilst the kinematic evaluation focused on kinematic parameters of the hooker and of the ball (as suggested by Sayers, 2011).

## Methods

### Participants

Twenty national level players (mean age 21.85 ± 4.98 years) from the Italian Rugby Federation (FIR) were recruited for this pilot study. All the enrolled subjects were rugby players with at least of 5 years of expertise in competition settings and an 8 sessions/week training regimen (see Table 1).

**Table 1:** Mean values (±SE) of the participants' expertise in rugby play, the number of throws hitting the target (Throws IN), and of the ball and hooker kinematic parameters acquired during PRE and POST evaluation epochs in Action Observation (AO) and CONTROL groups.

**Parameters**	**AO group**		**CONTROL group**	
**Expertise (years)**	6.70 ± 0.63		6.62 ± 0.65	
	**Pre**	**Post**	**Pre**	**Post**
Throws IN	8.50 ± 0.99	12 ± 0.92	6.90 ± 1.08	7.5 ± 1.05
**KINEMATIC OF THE BALL**				
Ball velocity (m/s)	8.88 ± 0.33	10.29 ± 0.55	9.41 ± 0.38	9.68 ± 0.42
Ball angle release (°)	30.23 ± 1.49	29.20 ± 1.38	33.13 ± 1.14	31.65 ± 0.83
Ball spinning (°)	37.44 ± 2.17	33.39 ± 1.19	36.51 ± 1.63	29.82 ± 1.60
**KINEMATIC OF THE HOOKER**				
Knee angle at the end of the backswing phase (°)	139.30 ± 6.99	133.10 ± 3.46	155.25 ± 3.35	156.90 ± 3.30
Knee angle at ball release (°)	169.50 ± 1.63	163.65 ± 2.30	166.75 ± 4.19	165.60 ± 3.81
Elbow angle at the end of the backswing phase (°)	62.25 ± 4.10	70.20 ± 3.90	58.85 ± 2.49	60.50 ± 2.46
Elbow angle at ball release (°)	116.25 ± 4.80	120.40 ± 5.87	128.75 ± 5.23	129.20 ± 5.34
Arm's mean velocity (m/s)	5.42 ± 0.07	6.43 ± 0.05	5.28 ± 0.04	5.53 ± 0.04

Participants were randomly divided into two groups according to a block randomization method: the action observation group (AO) (*n* = 10), in which participants carried out a combination of observational training with conventional lineout training, and the CONTROL group (*n* = 10) in which participants performed only the conventional lineout training.

The recruitment process consisted of a medical evaluation to assess their good health and the absence of any contraindication to participation in the experimental protocol. During the intervention period the team doctor daily visited athletes in order to ensure the absence of injuries that could compromise the participation in the project.

All participants were naive to the purpose of the experiment as well as about the AO technique as training method, and gave informed and written consent for participation in the study prior to testing. The study was approved by the Ethics Committee of the University of Genoa and was conducted in accordance with the 2013 revision of the Declaration of Helsinki on human experimentation.

Expertise in rugby play was comparable between the two groups, as stated by the results of the Mann-Whitney non-parametric test (*Z* = 0.76, *p* = 0.41).Post-randomization tests performed after the evaluation of the kinematic and performance parameters at baseline (results are not reported here) confirmed that the characteristics of the two groups were homogeneous (see Measures for description of the variables and Table 1 for parameters values at baseline).

### Experimental Protocol

AO and CONTROL groups did not train together, and were blind about the kind of training methodology the other group was performing, and about the performance obtained by the athletes of the other group. The AO group was required to observe a video-clip (duration 4 min and 58 s, see Supplementary Video 1) showing 60 repetitions of a lineout throwing performed by an expert model toward a target positioned at 7 m distance, and at 3.28 m height from the ground.

The video-clip was displayed from a side view in order to appreciate the hooker's posture and his throwing technique together with the ball kinematics. The throw was executed with an overhead action and it was fully two-handed, maintaining a stationary base of support throughout the throwing action with a semi-tandem foot configuration. Spatiotemporal characteristics of the action were the following: global lineout throwing duration: 2.47 s; preparation: duration 0.84 s; backswing: duration 0.76 s, mean velocity 1.32 m/s; forward throw: duration 0.16 s, mean velocity 6.68 m/s. Kinematic of the ball: mean velocity 12.01 m/s, ball angle release 39°, and ball spinning 42°. Kinematic of the hooker at the end of the backswing phase: knee angle 130° and elbow angle 45°; at the ball release: knee angle 155° and elbow angle 133°. Hooker's arm mean velocity 6.49 m/s (see Measures for description of the variables). Each throw was followed by the observation of a fixation cross lasting 2.5 s. After watching the video-clip, the athletes in the AO group performed the lineout training, consisting of six repetitions x five blocks of throws (total = 30) toward a target placed at 7 m distance away. The CONTROL group was asked to perform the same number of throws without watching the video.

**Figure 1:**
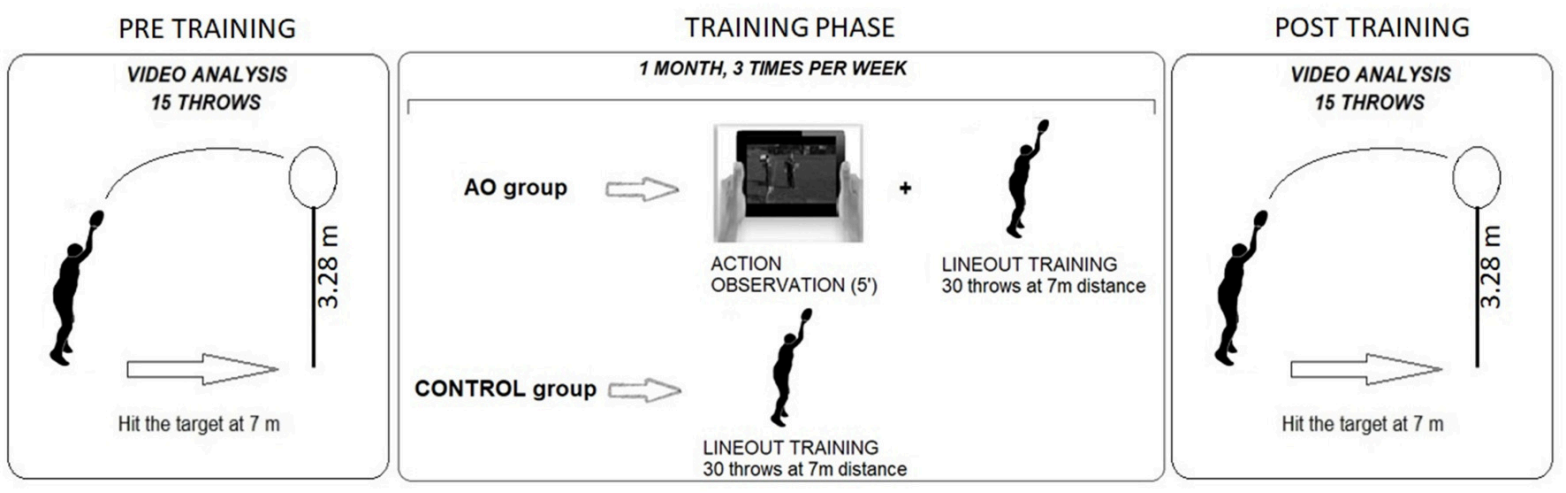
Experimental protocol. Twenty participants were enrolled in this study and assigned to two groups, Action Observation (AO) group and CONTROL group. The two groups were involved in two different lineout trainings (TRAINING PHASE) that lasted 1 month (three training sessions per week); the AO group observed a 5 min video showing 60 repetitions of a lineout throw performed by an expert model and then performed 30 lineout throws toward a target positioned 1 m distant at 3.28 m from the ground. Participants in the CONTROL group executed the 30 lineout throws. Before (PRE TRAINING) and after (POST TRAINING) the training phase, all participants participated to a testing session during which they were filmed while executing 15 lineout throws in order to evaluate their movement kinematics and ball kinematics.

Both groups performed the training in the conventional outdoor rugby field. The experimental protocol (see Figure 1) lasted 4 weeks and the athletes were trained three times per week (total number of sessions = 12). Each lineout training session lasted about 20 min.

### Measures

**Figure 2:**
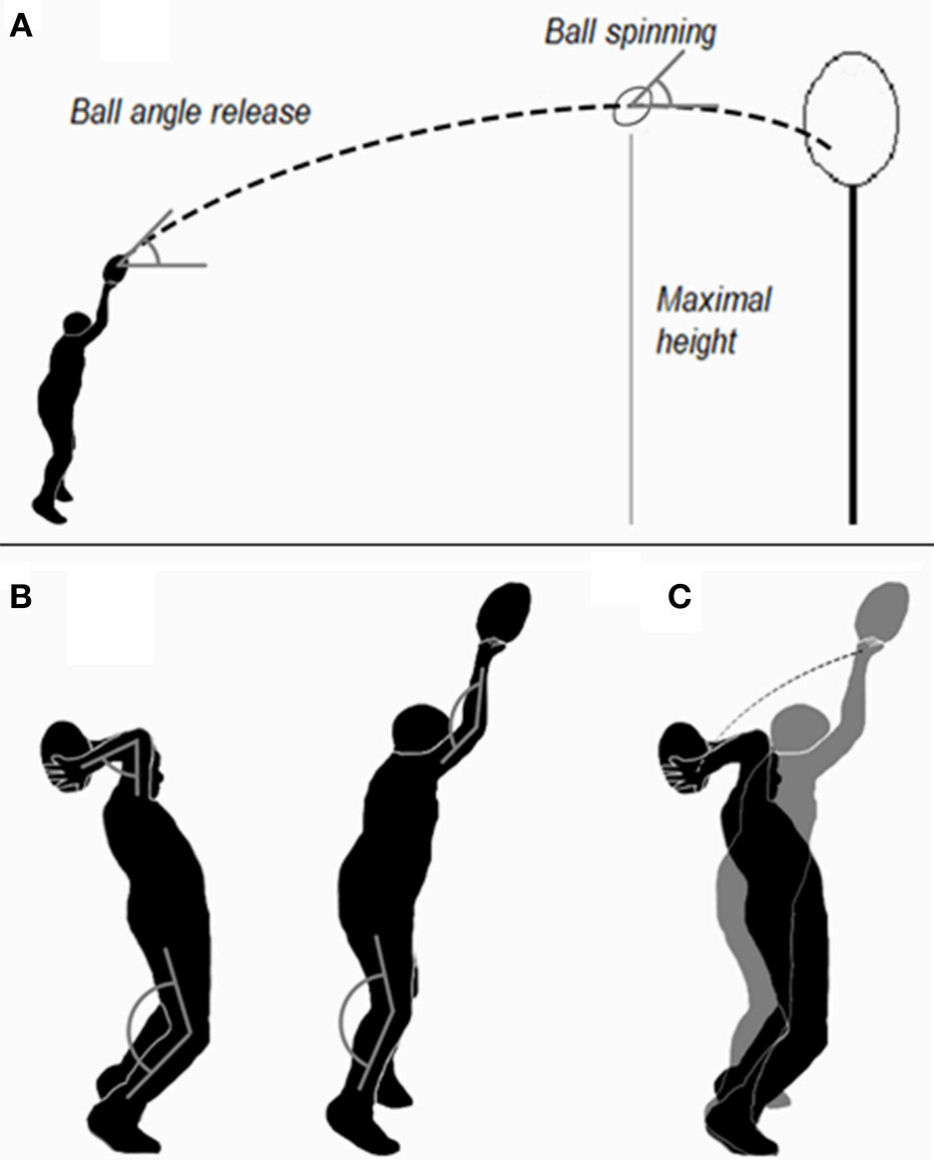
Kinematic measures. **(A)** indicates the ball angle computed at release (ball angle release), ball spinning, and ball velocity computed at maximal trajectory height; **(B)** shows hooker's elbow and knee angles at the end of the backswing phase and at ball release; **(C)** displays Hooker's arm trajectory used to compute the mean velocity value.

Skill performance and kinematic parameters were evaluated in an outdoor rugby field before (PRE) and after (POST) the intervention period. During the evaluation, athletes were asked to perform 15 throws, trying to hit a target located 7 m away from their position, and at 3.28 m height from the ground (target dimensions: 0.4 m). In all trials, participants were asked to throw, as accurately as possible, using their usual throwing technique with the aim of hitting the target. Each throw was filmed by a video camera. Each video was analyzed by means of Kinovea software (Kinovea, 0.8.15; Copyright 2006–2011, Joan Charmant & Contrib). The throwing accuracy was evaluated as the number of throws hitting the targets (Throws IN). The kinematic evaluation focused on: (a) the kinematic parameters of the ball, namely ball velocity computed in the point corresponding to its trajectory maximum height, ball angle release (computed as the angle between the horizontal line and the line connecting the two extreme points of the ball) (Trewartha et al., 2008), and ball spinning (computed as the angle defined by the horizontal line tangent to the ball trajectory when it reached the maximal height and the line connecting the two extreme points of the ball), (b) the hooker's elbow and knee angles at the end of the backswing and at the ball release (Sayers, 2011), and the hooker's arm mean velocity during the forward throw (from the end of the backswing to the ball release). A researcher, blinded to the experimental protocol, performed all the video analysis at each time point (PRE and POST) (see Figure 2).

### Statistical Analysis

Throws IN, ball velocity, ball angle release, ball spinning, hooker's elbow and knee angles, and hooker's arm mean velocity were normally distributed according to the results of Shapiro–Wilk tests.

Mixed-model ANOVAs, with GROUP (2 levels: AO and CONTROL), as between subject factor, and TIME (2 levels: PRE and POST), as within subject factor, were applied to the Throws IN, ball speed, ball angle release, ball spinning, knee, and elbow internal angles at the end of the backswing phase and at ball release, and hooker's arm mean velocity during the forward throw. Significant interactions were analyzed by means of Bonferroni *post hoc* comparison.

Furthermore, regressions analysis (Pearson's correlation) was used to test the existence of a linear relationship between the variation of the number of throws hitting the target (Δaccuracy = Throws IN_POST_ – Throws IN_PRE_) and the changes in the value of the parameters that resulted to evolve differently in the two groups after the training. The same analysis was applied to test the relationship between the changes in hooker's arm velocity and ball velocity since a higher velocity of the hooker's arm might determine a higher velocity of the ball.

Data are presented as mean ± standard error (SE).

## Results

### Throwing Accuracy

**Figure 3:**
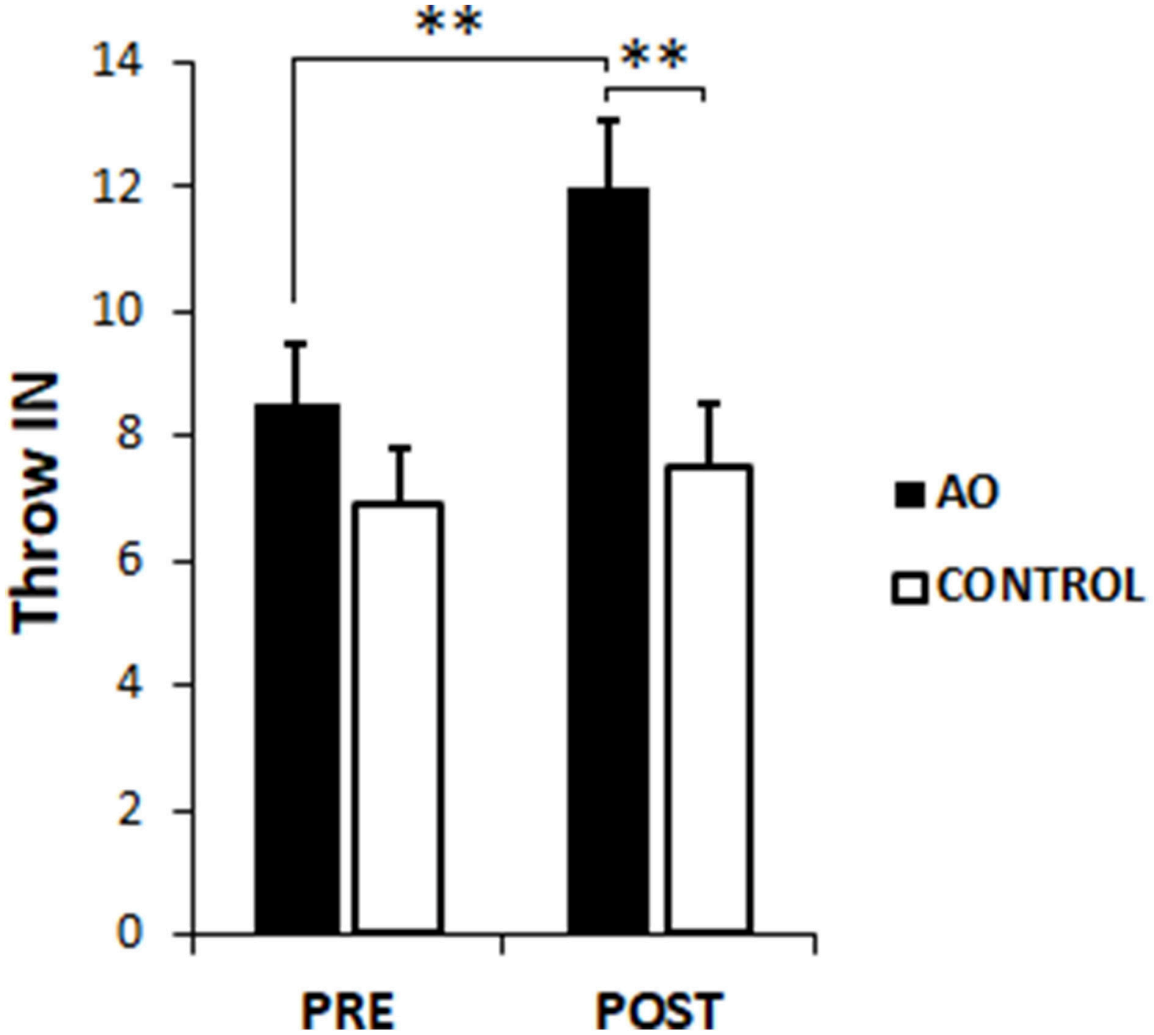
Mean throwing accuracy. Number of throws falling into the target computed before (PRE) and after (POST) the training phase, in action observation group (AO, black column) and CONTROL group (white column). Bars indicate the SE. ^**^p < 0.01.

The throwing accuracy (Throws IN) for the two groups and in the two evaluation epochs are represented in Figure 3. The result of the mixed-model ANOVA showed a significant main effect of TIME [PRE = 7.64 ± 0.72, POST = 9.71 ± 0.70 m/s, *F*_(1, 18)_ = 10.50, *p* < 0.01, η^2^ = 0.33] and of GROUP [AO group = 10.15 ± 0.83 m/s, CONTROL group = 7.20 ± 0.95, *F*_(1, 18)_ = 5.47, *p* < 0.05, η^2^ = 0.21]. Furthermore, TIME^*^GROUP interaction [*F*_(1, 18)_ = 4.68*, p* < 0.05, η^2^ = 0.21] and *post hoc* comparisons revealed a significant increased Throws IN in the AO group after the intervention period [PRE vs. POST, mean difference = 3.54, *p* = 0.0009, 95% CI (1.79, 5.29)], whilst no significant changes were observed in the CONTROL group [PRE vs. POST mean difference = 0.60, *p* = 0.61, 95% CI (−1.40, 2.60)]. Furthermore, while PRE epoch Throws IN of the two groups were not statistically different [mean difference = 1.49, *p* = 0.31, 95% CI (−4.48, 1.51)], their values in the AO group were significantly higher than those in the CONTROL group at POST [mean difference = 4.42, *p* = 0.004, 95% CI (1.53, 7.31)].

### Kinematic of the Ball

**Figure 4:**
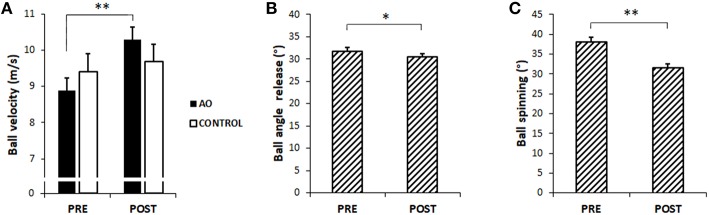
Kinematic of the ball. Mean values and SE of **(A)** ball velocity (m/s), **(B)** ball angle release (°), and **(C)** ball spinning (°) before (PRE) and after (POST) the training phase. The significant interaction is represented for ball velocity, while the main effect of TIME is displays for ball spinning and angle release. Black column indicates AO group and white column indicates CONTROL group. Dashed columns indicate the mean values of the two groups. ^*^p < 0.05, ^**^p < 0.01.

Ball velocity, ball angle release, and ball spinning values are represented in Figure 4. Concerning ball velocity, a significant main effect of TIME was found [PRE = 9.14 ± 0.25 m/s, POST = 9.99 ± 0.35 m/s, *F*_(1, 18)_ = 9.75, *p* < 0.01, η^2^ = *0.35*]. Furthermore, TIME^*^GROUP interaction was found [*F*_(1, 18)_ = 4.42, *p* < 0.05, η^2^ = 0.20] and the following *post hoc* tests showed a significant increase of its values only in the AO group [PRE vs. POST; AO group, mean difference = 1.41, *p* = 0.002, 95% CI (0.61, 2.21); CONTROL group, mean difference = 2.78, *p* = 0.48, 95% CI (0.53, 1.08)]. No significant differences appeared between groups in both evaluation epochs [AO vs. CONTROL: PRE, mean difference = 0.52, *p* = 0.31, 95% CI (−1.58, 0.54); POST mean difference = 0.61, *p* = 0.39, 95% CI (−0.85, 2.08)].

A significant effect of TIME was shown by the result of the ANOVA on ball angle release [*F*_(1, 18)_ = *5.01, p* < 0.05, η^2^ = 0.22] due to a significant decrease in angle value in both groups after the training period (PRE 31.68 ± 0.94°, POST 30.43 ± 0.81°).

At last, ANOVA on ball spinning showed a significant effect of TIME [*F*_(1, 18)_ = 42.80*, p* < 0.0001, η^2^ = 0.70]; after the training period ball spinning value of both groups significantly decreased (PRE 37.98 ± 1.36°, POST 31.61 ± 0.99°).

### Kinematic of the Hooker

The hookers of both groups executed a fully two-handed throw with an overhead action and they maintained a stationary base of support with a semi-tandem foot configuration and they did not change the throwing features between timepoint 1 and timepoint 2.

The two training protocols did not result in significant changes in elbow angles and in knee angles at the end of the backswing and at ball release.

**Figure 5:**
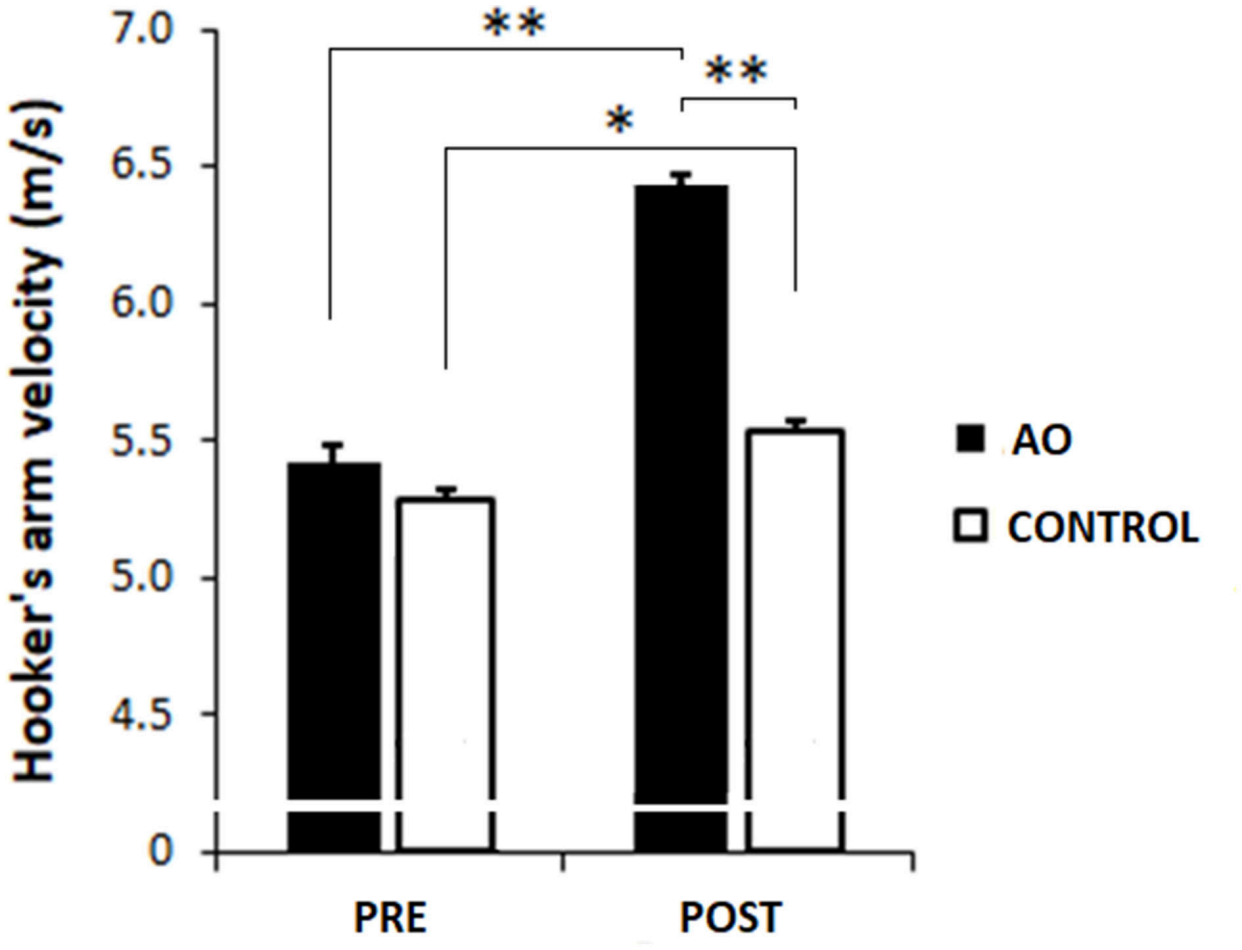
Kinematic of the hooker: mean hooker's arm velocity (m/s). Mean values of hookers' arm velocity before (PRE) and after (POST) the training phase in action observation group (AO, black column) and CONTROL group (white column). Bars indicate the SE. ^*^p < 0.05, ^**^p < 0.01.

The analysis on the hooker's arm mean velocity showed a significant main effect of TIME [PRE = 5.35 ± 0.04 m/s, POST = 5.98 ± 0.32 m/s, *F*_(1, 18)_ = 207.68, *p* < 0.0001, η^2^ = 0.92) and of GROUP [AO group = 5.92 ± 0.04 m/s, CONTROL group = 5.41 ± 0.04 m/s, *F*_(1, 18)_ = 85.11, *p* < 0.0001, η^2^ = 0.83). Furthermore, a significant TIME^*^GROUP interaction [*F*_(1, 18)_ = 74.19, *p* < 0.0001, η^2^ = 0.81]. Hooker's arm velocity at baseline was comparable between groups [PRE; AO group vs. CONTROL group, mean difference = 0.14, *p* = 0.10, 95% CI (−0.03, 0.30)]. Then, *post hoc* comparisons revealed a significant increase of arm's velocity in both groups [PRE vs. POST; AO group, mean difference = 1.02, *p* = 0.0001, 95% CI (0.89, 1.15); CONTROL group, mean difference = 0.26 *p* = 0.001, 95% CI (0.13, 0.39)]. Furthermore, AO group showed a significantly higher velocity than the CONTROL group in POST evaluation epoch [mean difference = 0.90, *p* = 0.0002, 95% CI (0.77, 1.03)] (Figure 5).

### Regression Analysis

**Figure 6:**
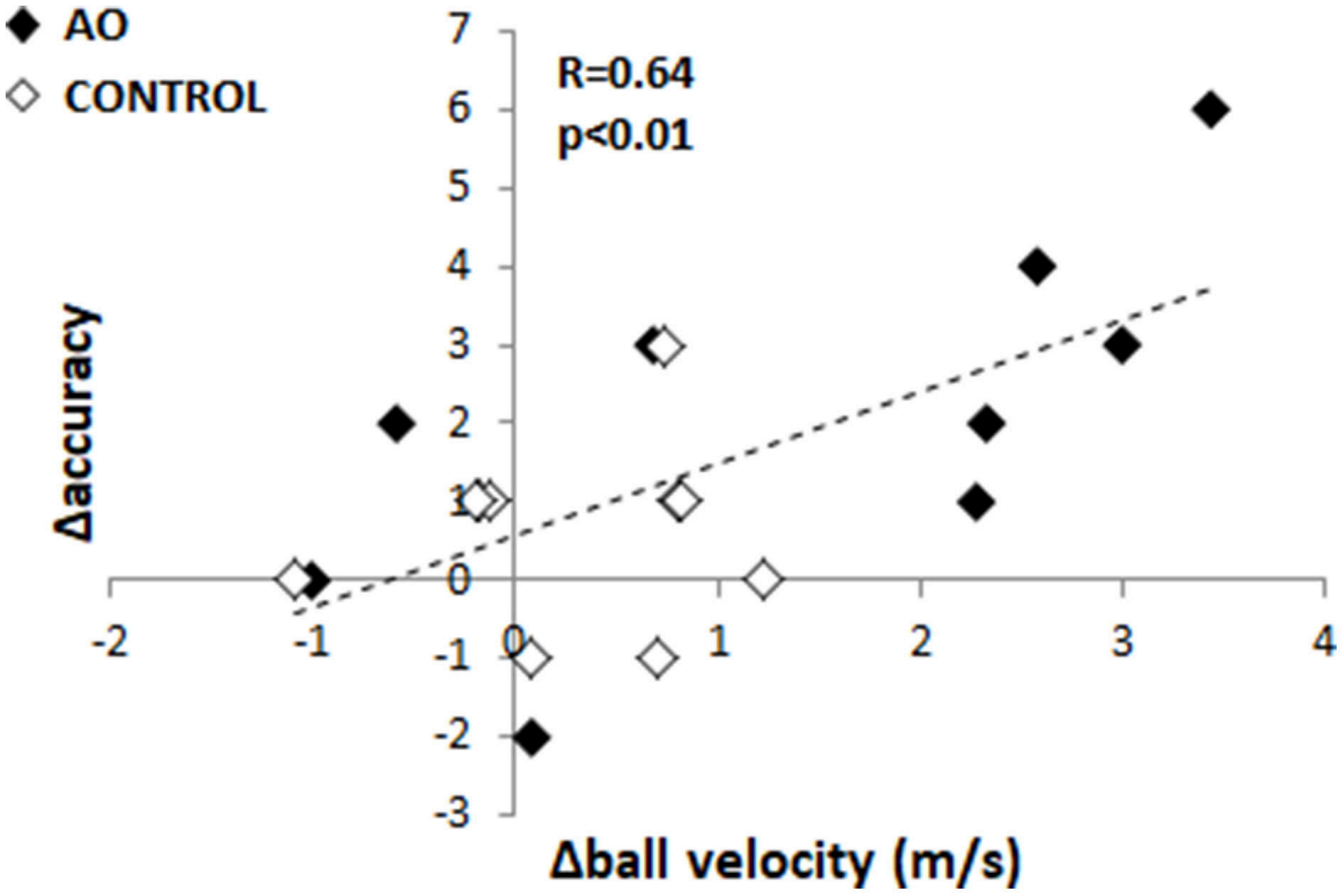
Linear relationship between changes in throwing accuracy (Δaccuracy) and in ball velocity (Δball velocity). Black and gray diamonds indicate data of action observation (AO) group and CONTROL group, respectively.

Pearson's correlation analyses were performed to test the relationship between Δ-accuracy and (1) the variation of mean ball velocity (Δ-ball velocity = ball velocity_POST_ – ball velocity_PRE_), (2) hooker's arm velocity increase. Since the Δ-accuracy value of one subject in the AO group came out to be an outlier (Δ-accuracy was higher than mean values ±2^*^SE), we removed it from this specific analysis. A significant positive correlation appeared only between ball velocity and the number of target hit (*R* = 0.64*, p* < 0.01) (Figure 6). A trend toward the significance appeared between the increase in hooker's arm velocity and ball velocity (*R* = 0.40*, p* = 0.07).

## Discussion

The aim of this study was to investigate in elite rugby players whether a combination of AO and conventional training is able to improve kinematic parameters of the lineout throwing and the outcome of the motor performance, more than a conventional training alone.

To this aim, 20 elite rugby players were recruited and randomly divided into two groups. One group received an observational training, by watching 60 repetitions of a video-clip showing an expert model performing a lineout throw, immediately followed by a conventional lineout training (AO group), and the other group was involved only in a conventional training (CONTROL group). A significant improvement in throwing accuracy (Throws IN) was found in AO group, whilst no significant difference was observed after the conventional training alone. As concerns kinematic parameters, the hooker's arm velocity, during the forward swing phase, significantly increased in both groups, with higher values in AO group. Furthermore, the AO group showed a significant increase of the ball velocity, whilst in both groups the ball angle release, and the ball spinning decreased significantly. In both groups, no changes were observed in knee and elbow angles at the end of the backswing phase and at ball release.

A number of studies in neuroscience and rehabilitation demonstrated that AO contributes to the learning of a wide variety of tasks (Stefan et al., 2005; Wulf and Mornell, 2008; Sale and Franceschini, 2012; Buccino, 2014; Avanzino et al., 2015; Abbruzzese et al., 2016; Lagravinese et al., 2017), but until now its effectiveness is not yet fully demonstrated in sports. The rationale of using AO as training technique in sport is that the neural mechanisms activated by AO are similar to those activated during physical practice (Badets and Blandin, 2004; Rizzolatti and Craighero, 2004; Badets et al., 2006). This hypothesis is well-supported by neuroimaging studies that indicate the existence of an “action observation network” that engages the observer in processes similar to those involved during physical practice (Buccino et al., 2001; Cisek and Kalaska, 2004; Cross et al., 2009; Dushanova and Donoghue, 2010) and this effect is enhanced when movement observation is combined with an immediate training (Celnik et al., 2006, 2008; Stefan et al., 2008).

A facilitation of learning when physical practice was combined with AO was observed in different sports (Guadagnoli et al., 2002; Hodges et al., 2005; Breslin et al., 2006; Hayes et al., 2007; Kim et al., 2011; Bouazizi et al., 2014; Sakadjian et al., 2014; D'Innocenzo et al., 2016). All these studies pointed out the beneficial role of AO training in improving the motor performance and the athletes' movement kinematics. None of them investigated the effects of AO applied during a complex motor skill, such as the lineout in rugby, in which this gesture represents a key aspect of game play that is unique to this sport. In fact, it was calculated that during the International Rugby Union competitions (World Rugby Game Analysis, 2015) the lineout was played ~26 times per match, and the ability to maintain the ball possession during the lineout is a discriminative factor between losing and winning teams (Ortega et al., 2009). Successful lineout throws require the ball to be delivered accurately to the hands of a teammate over distances between 5 and 18 m.

Despite the importance of lineout throwing in rugby game play, only recently biomechanical analyses of this gesture have been presented in the scientific literature (Sayers, 2005, 2011; Trewartha et al., 2008). In particular, Trewartha et al. (2008) described the link between the kinematics of the lineout and the throwing accuracy. They concluded that consistent upper limb movement patterns, both in time and range of motion, were key elements for improving the accuracy of the throwing action. Consistently with these authors, our results showed that the significant improvements found in AO group in throwing effectiveness toward a target at 7 m were accompanied by significant increases in both hooker's arm velocity and ball velocity, confirming that these kinematic variables represent two key aspects of the lineout throws over short distances (5–7 m), i.e., typically throws that travel to the front of a rugby lineout. Furthermore, throwing accuracy and ball velocity have been here demonstrated to correlate with each other, reinforcing the conclusion that the increase in ball velocity contributes to improvements in throwing effectiveness. Interestingly, our results showed that a trend, although not significant, existed between the increase in hooker's arm velocity during the forward throw and the increase in ball velocity: namely, the higher the hooker's arm velocity, the higher the ball velocity. Another study performed in subjects throwing a ball toward a target with maximal velocity using a backhanded reverse baseball pitch, suggested that the model's relative motion patterns act as a rate enhancer, and demonstrated that ball velocity increased in tandem with new motor coordination patterns (Horn et al., 2007). Concerning the hooker's arm velocity, the statistical analysis showed a significant increase of the value of this parameter in both groups toward that of the expert model, but the increase was significantly higher in the AO group. Therefore, it can be hypothesized that AO training evoked a sort of implicit imitation mechanism (Bisio et al., 2010; Avanzino et al., 2015; Lagravinese et al., 2017) of the temporal aspect of both the hooker's movement and the ball velocities, indicating that AO might be fruitful in significantly improving the movement timing more than other characteristics.

Concerning other kinematic parameters, we showed that the ball angle release and the spinning of the ball significantly decreased in both groups, equally resulting in a more precise and clean throwing trajectory toward the jumper.

Finally, no significant changes in knee angles were observed in both groups. A possible explanation of this outcome can take into account the results of biomechanical analyses of lineout gesture (Sayers, 2011), where it has been highlighted that for throws traveling for short distances (as those required in our protocol) the key factors for improving accuracy are time and range of upper limb movement patterns (i.e., higher velocity and reduced ball angle release), whereas the involvement of lower limbs acquires increasing importance when throws travel long distances (15–18 m). A different explanation for the lack of effects of AO on hooker's upper and lower limbs angles could be that during AO training rugby players' attention was not directed toward a particular aspect of the video, but they extracted the information that they think might be more useful to improve the final outcome (i.e., the throwing accuracy), which, in that case, resulted to be ball velocity, ball angle release and hooker's arm velocity.

### Limitations

In this study, we focused on a rugby specific motor gesture, namely lineout throwing. We acknowledge that our findings are related primarily to this motor skill, but other recent studies (Breslin et al., 2006; Hayes et al., 2007; Hodges et al., 2007; Horn et al., 2007), supporting our results, suggest the possible efficacy of AO training in other complex motor tasks in sport. Another limitation concerns the small number of subjects recruited in this study; however, in the literature, often pilot studies, testing AO training, have been conducted and published with a small number of participants (e.g., Sakadjian et al., 2014; Fukuhara et al., 2018; Unenaka et al., 2018). Although we are aware that the involvement of a larger cohort would have strengthened the results obtained, however, giving the homogeneity of the two groups in terms of both expertise, and performance and kinematic parameters at baseline (T0), we were able to detect clear differences with an effect size from small to large values, implying the number of subjects was sufficient for providing significant results from this pilot study. Another criticism is the lack of a second control group who observed a neutral video that does not contain information regarding the lineout throwing. Often the observation of landscapes' video is used as a control condition. However, under our experimental conditions, it should not represent a more appropriate method than that used in this study, since it should be very difficult to engage a higher level of attention, especially during a sport training.

## Conclusion

This study provides new information on the effects of combining AO and immediate physical practice in both learning motor skills and improving motor performance in sports. Particularly, we showed that this combined training program is more effective than a conventional training alone in enhancing the performance of elite rugby players during a complex motor skill, such as the lineout. This led to an improvement in throwing accuracy and to significant changes in hooker's and ball's kinematic variables.

The present findings provide further evidence concerning the fruitful application of AO during sports training, and support AO as methodology to potentiate the effectiveness of a conventional lineout training. This study provides an innovative and easy-to-apply model of lineout training program; indeed, the video of the model's action seen directly on the field offers the athletes the opportunity to perform their usual physical training immediately after the observation, thus increasing the possibility of a short-term consolidation of the observed action. The results of this study can assist coaches in designing specific lineout training programs, proposing an innovative and fruitful approach to cognitive training, that could be easily applied on the field. Finally, this study, together with others applying neuroscientific methodologies during sport training, may also encourage sport scientists and coaches to collaborate toward an evidence-based coaching.

## Ethics Statement

The experimental protocol was carried out in agreement with legal requirements and in accordance with the 2013 revision of the Declaration of Helsinki on human trials.

## Author Contributions

EF, EP, and PR contributed conception and design of the study and critically discussed the results. LS and VF performed the experimental study. AB, LP, and VF organized the database and performed the statistical analysis. EF and AB wrote the first draft of the manuscript. All authors contributed to manuscript revision, read, and approved the submitted version.

### Conflict of Interest Statement

The authors declare that the research was conducted in the absence of any commercial or financial relationships that could be construed as a potential conflict of interest.

## Supplementary Material

Supplementary Video 1 Video observed by athletes in Action Observation group. The video-clip, displayed from a side view, showed an expert model performing a line out throw at 7 m distance.
